# *De novo* design of a stapled peptide targeting SARS-CoV-2 spike protein receptor-binding domain†

**DOI:** 10.1039/d3md00222e

**Published:** 2023-07-31

**Authors:** Ravindra Thakkar, Dilip K. Agarwal, Chathuranga B. Ranaweera, Susumu Ishiguro, Martin Conda-Sheridan, Natasha N. Gaudreault, Juergen A. Richt, Masaaki Tamura, Jeffrey Comer

**Affiliations:** a Department of Anatomy & Physiology, Kansas State University College of Veterinary Medicine Manhattan Kansas USA ravithakkar@ksu.edu; b Department of Material Science and Engineering and NUANCE Center, Northwestern University Evanston Illinois USA; c Department of Medical Laboratory Sciences, General Sir John Kotelawala Defense University Colombo Sri Lanka; d Department of Pharmaceutical Sciences, College of Pharmacy, University of Nebraska Medical Center Omaha Nebraska USA; e Department of Diagnostic Medicine & Pathobiology, Kansas State University College of Veterinary Medicine Manhattan Kansas USA

## Abstract

Although effective vaccines have been developed against SARS-CoV-2, many regions in the world still have low rates of vaccination and new variants with mutations in the viral spike protein have reduced the effectiveness of most available vaccines and treatments. There is an urgent need for a drug to cure this disease and prevent infection. The SARS-CoV-2 virus enters the host cell through protein–protein interaction between the virus's spike protein and the host's angiotensin converting enzyme (ACE2). Using protein design software and molecular dynamics simulations, we have designed a 17-residue peptide (pep39), that binds to the spike protein receptor-binding domain (RBD) and blocks interaction of spike protein with ACE2. We have confirmed the binding activity of the designed peptide for the original spike protein and the delta variant spike protein using micro-cantilever and bio-layer interferometry (BLI) based methods. We also confirmed that pep39 strongly inhibits SARS-CoV-2 virus replication in Vero E6 cells. Taken together these data suggest that a newly designed spike protein RBD blocking peptide pep39 has a potential as a SARS-CoV-2 virus inhibitor.

## Abstract 2

Binding configuration of a *de novo* stapled peptide on SARS-CoV-2 spike protein, as predicted by molecular simulation. Stapled residues enhance peptide stability while interacting residues engage key amino acids on the protein receptor-binding domain.

## Graphical abstract

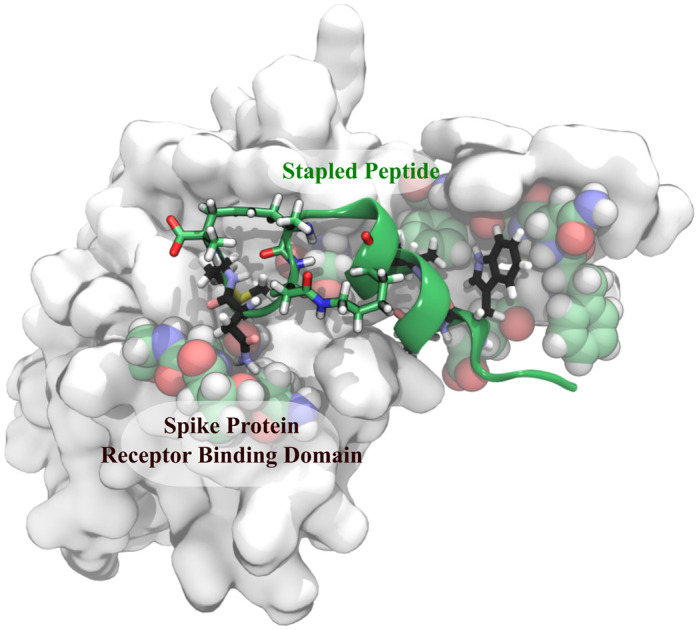

## Introduction

A new infectious respiratory disease was reported in Wuhan, China, in December 2019.^1^ This disease, termed COVID-19 by the World Health Organization, was identified as being caused by a novel coronavirus and has been a major threat to global public health and the economy. As of today, more than 453 million people have been infected, and 6 million deaths have been reported due to the COVID-19 pandemic.^2^ Even after the development of effective vaccines, this disease continues to cause disability and death across the globe. Current treatments possess several shortcomings; for example, vaccination is less effective for immuno-compromised patients, and vaccine hesitancy and limited vaccine distribution have contributed to sizeable populations remaining unvaccinated.^3^ Cases of reinfection in patients who had fully re-covered from COVID-19 have been reported.^4,5^ Antibodies against SARS-CoV-2 seem to have a short life span, and the titer often decreases in a few months after the onset of symptoms.^6^ Inconsistency in the ability to produce effective antibodies against the spike protein has been observed in various patients.^7,8^ Given all this, a set of effective antiviral drug could complement vaccination and be a powerful tool in the continuing fight against COVID-19.^9^ Indeed, treatments such as nirmatrelvir/ritonavir (Paxlovid), which consists of a protease inhibitor (nirmatrelvir) and an CYP3A4 inhibitor (ritonavir), have demonstrated efficacy in reducing rates of mortality and hospitalization.^10^

The virus causing COVID-19 exhibits 80% sequence similarity with SARS-CoV, a virus that emerged in 2002–2003, which is why the virus has been dubbed SARS-CoV-2.^11^ These coronaviruses enter the host cells by binding to the angiotensin converting enzyme 2 (ACE2), a receptor protein on the surface of human cells. The receptor binding domain (RBD) of the prominent viral spike protein is responsible for this binding.^12^ The spike protein of SARS-CoV-2 has evolved to bind ACE2 with high affinity and is an important factor in its high contagiousness.^13^ X-ray crystallography of the complex between the SARS-CoV-2 spike protein and ACE2 has revealed some of the key amino acid residues where the ACE2 protein binds and provides valuable information needed to design therapeutic drugs that can block the spike protein surface, preventing entry of the coronavirus into the host cell.^14^

**Fig. 1:**
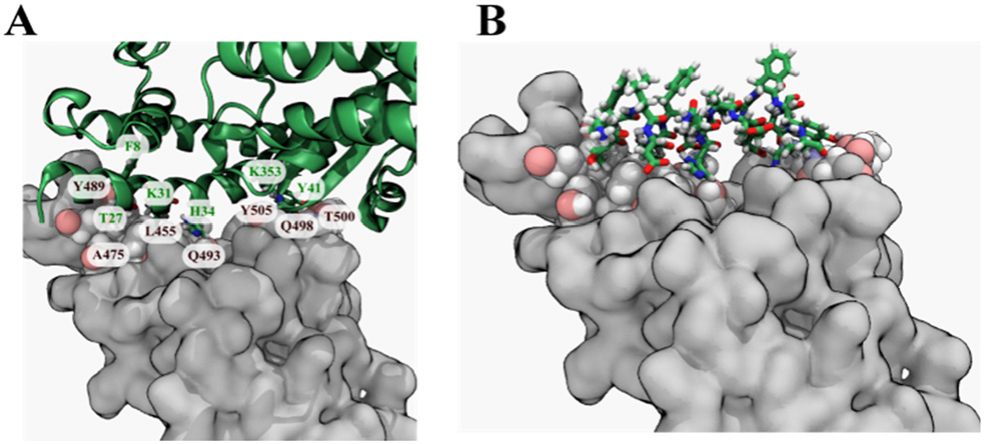
Selection of the template peptide from ACE2. (A) Crystal structure of the spike protein RBD and ACE2 protein complex (PDB ID 6LZG). The spike protein RBD is shown as a grey surface, while ACE2 is shown in a green secondary structure representation. Key residues in the contact interface between the spike protein RBD and are labelled (brown, spike protein RBD; green, ACE2). (B) A fragment of the ACE2 protein containing amino acid numbers 26–42 was selected as a template for the peptide design.

Several small drug molecules have been predicted to target the spike protein RBD by computational studies.^15,16^ However, small molecules are, by nature, too small to occupy the entire portion of the RBD surface that forms the interface with ACE2 (Fig. 1A). Synthesis of custom peptides has become routine and commercialized. While commercial synthesis of novel conventional small-molecule drugs exists, peptide synthesis remains more accessible.^17^ Previous studies have reported that linear therapeutic peptides based on the human ACE2 alpha-1 helix have been shown to inhibit the interaction between ACE2 and the spike protein RBD.^18–20^ Furthermore, linear peptides have poor conformational and proteolytic stability. Linear peptides are flexible and, therefore, the entropic cost for adopting a more restricted conformation when bound to the target is high.^21^ The introduction of chemical crosslinks into peptides, such as those referred to as “staples”, helps to maintain the bound conformation and reduce the entropic cost for binding. Some stapled peptides that bind to the SARS-CoV-2 spike protein and its mutants have been developed, but they did not prevent virus internalization into host cells.^22^ These studies also did not address the delta and omicron variants of SARS-CoV-2, which have significantly mutated spike proteins that are less sensitive to host antibodies from recovered or vaccinated individuals.

This study presents the *de novo* computational design of a stapled peptide and evaluation of its binding activity with the spike protein RBD and those of variants by molecular simulation and label-free binding techniques like micro-cantilever and bio-layer interferometry.

## Results and discussion

### Selection of the starting template for the peptide design

The experimentally determined X-ray crystal structure of a complex of the ancestral strain spike protein RBD and human ACE2 protein (PDB ID: 6LZG) provided a high-resolution atomic model (2.50 Å). The α1-helix of the hACE2 protein occupies a long and flat interface of the spike protein RBD, which is reported to have an area of around 225 Å^2^.^23^ Residues Thr27, Phe28, Lys31, His34, Try41 and Lys353 of ACE2 protein were identified as key residues using KFC2, the knowledge-based protein–protein interface prediction webserver web-server (Fig. 1A).^24^ Hence, residues 26 to 42 from the α1–helix of the hACE-2 can provide an appropriate starting template (Fig. 1B).

### Docking and sequence modification to optimize peptide

**Fig. 2:**
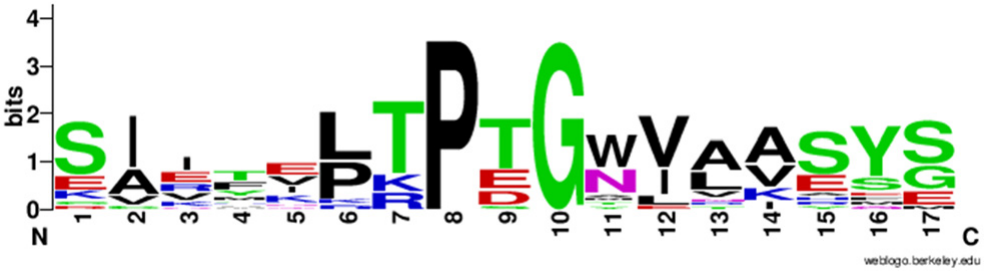
Graphical representation of the frequency of residues at specific positions in the peptide. The height of symbols represents the relative frequency for the occurrence of the residue in our Rosetta optimization. Created with WebLogo.

We applied the structure based FlexPepDock protocol to design a peptide that binds the spike protein RBD. FlexPepDock performs a large-scale search of the backbone conformational space. We selected poses that were both low energies according to Rosetta and had structural similarity with the template peptide to create a peptide that binds to the RBD in the same location as the α1-helix of hACE2 protein. The poses were selected by plotting the docking score *vs.* RMSD (Fig. S1†). To increase the affinity of selected poses for the spike protein RBD, we applied a protein design protocol of Rosetta that performs sidechain and rotamer optimization to make the estimated binding energy more favourable. The selection of amino acids for substitution was unbiased (all 20 canonical proteinogenic amino acids were available). Although multiple rounds of optimization gave a variety of sequences, certain positions on the peptide favoured particular residues. For example, the initial backbone structure was consistent with only proline and glycine at the 8th and 10th positions, respectively. The 2nd, 12th, 13th, and 14th positions were dominated by hydrophobic residues. Fig. 2 shows the occurrence of residues at each position in the peptide.^25^

### Estimation of the binding free energy of optimized peptides

We performed all-atom explicit solvent molecular dynamics simulations for 41 optimized peptide structures with the spike protein RBD. We set up the simulations such that a simulation would terminate if the peptide structure deviated (in RMSD of Cα atoms) more than 15 Å from the starting configuration. We then estimated binding free energy calculation for all simulations by the MMGBSA method.^26,27^ Interestingly, the simulated time for which the peptide remained bound (RMSD <15 Å) was more correlated with the MMGBSA estimation of the binding free energy than the score assigned by the Rosetta (see Table S1†).

### Effect of stapling on the backbone conformation of the peptide

**Fig. 3:**
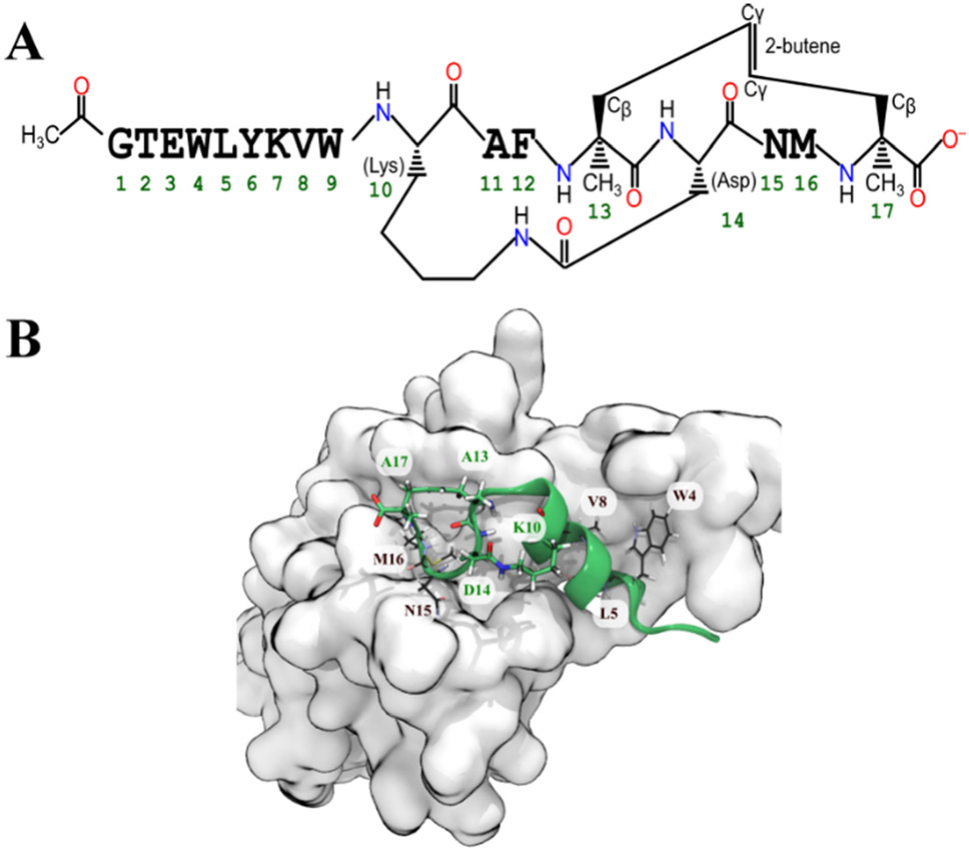
(A) Schematic diagram shows the chemical structure of the stapled peptide, pep39 (B) favourable configuration of the optimized peptide (green cartoon) on the spike protein RBD (grey surface). The chosen configuration was the simulation frame with the lowest MMGBSA energy. Some residues whose sidechains did not make contact with the protein were stapled (green labels), so as to improve the conformational stability of the peptide while not interfering with its interactions with residues of the spike protein RBD (black labels).

Based on the MMGBSA score we shortlisted nine out of the 41 optimized peptides. For all cases, the most favoured binding pose (according to the GBSA free energy function) had drifted substantially from the initial pose. To maintain the α-helical structure of the peptide and presumably reduce the entropic cost of binding, we added a propene staple between Val13 and Ser17, and covalently linked the carboxylic acid group of Asp14 (after making a E14D mutation) to the primary amine of Lys10 creating an amide linkage (Fig. 3). We then performed an MD simulation followed by an MMGBSA calculation. Similar stapling strategies were applied to the other shortlisted peptides. Almost all the stapled peptides exhibited greater conformational stability and stayed longer in bound state. It was evident that the stapled form of the peptide pep39 exhibited a higher propensity towards the helical conformation compared to its unstapled counterpart, as illustrated in (Fig. S2†). In almost all cases, the MMGBSA binding free energy was more favourable for the stapled version than for the unstapled version (see Table S2†).

### Conformational flexibility of the receptor-bound peptide in the stapled and unstapled form

**Fig. 4:**
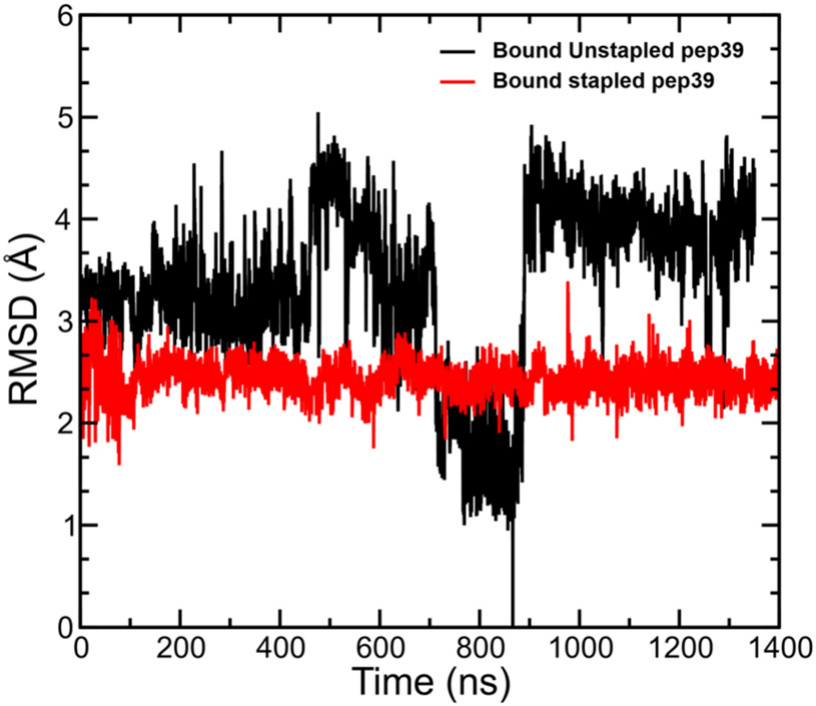
Conformational stability of unstapled and stapled versions of pep39. Deviation in the atomic positions of the stapled and unstapled versions of the peptide pep39 on the surface of the spike protein RBD. The RMSD is taken with respect to the unstapled structure with the most favorable GBSA binding free energy, which corresponds to the point with RMSD = 0 at *t* ≈ 870 ns.

We have analysed the MD simulation trajectories for the stapled and unstapled versions of peptides in the presence of spike protein RBD and measured the deviation in atomic positions between the stapled and unstapled peptides relative to the structure of the unstapled peptide with the most favourable Δ*G*^GBSA^_binding_. The stapled peptides in the bound state exhibited higher rigidity and lower mobility compared to their unstapled versions. A comparison of RMSD for the stapled peptide pep39 and its corresponding unstapled version is shown in Fig. 4.

### Absolute binding free energy calculation for pep39

The MMGBSA calculation gives an estimate for the binding affinity of pep39 for the spike protein RBD. However, we did not include the contribution from the conformational entropy and MMGBSA uses a continuum approximation for the hydration free energy, rather than explicit water molecules.^28^ Hence, to validate the binding affinity of pep39, an absolute binding free energy calculation by the geometric route was carried out using the BFEE plugin of VMD. The BFEE method explicitly includes the effects of individual water molecules and is more rigorously derived from statistical mechanics than the MMGBSA method. It has also been demonstrated to yield better agreement with experiment.^29^ The BFEE method includes entropy contributions, including conformational entropy of the peptide and configurational entropy of hydrating water molecules; however, for this reason, it requires extensive sampling of these degrees of freedom to yield correct results. This makes BFEE calculations computationally expensive. Based on results obtained from MMGBSA analysis, we applied a cut-off value of −30 kcal mol^−1^ MMGBSA free energy to shortlist 4 stapled peptide out of 15 for BFEE calculation. An extended ABF (eABF) calculation for each subprocess was run, and the overall simulation length was over 3.0 μs for each stapled peptide.^30^ The results obtained from the potential of mean force (PMF) calculations are given in Table S1.† Here, we will discuss the best performing stapled peptide, which we denote pep39, in further detail.

The contribution of each subprocess for absolute binding free energy is given in Table 1. The unbound stapled peptide does not always maintain the α-helical structure when unbound, while this structure is stable when bound to the spike protein RBD. These results are corroborated by the RMSD analysis of unbiased MD simulations of the stapled peptide while bound and unbound to the protein (Fig. S3†).

**Table 1:** Contribution of each subprocess to the absolute binding free energy calculation

Subprocess	Free-energy term	PMF (kcal mol^−1^)	Simulation time (ns)
1	Δ*G*_conform_	−17.94	1100
2	Δ*G*_*Θ*_	−0.25	18
3	Δ*G*_*Φ*_	−0.22	18
4	Δ*G*_*Ψ*_	−0.21	17
5	Δ*G*_*θ*_	−0.09	19
6	ΔG_*ϕ*_	−0.11	16
7	−*k*_B_*T* ln(S*I*C^0^)	−29.03	1181
8	Δ*G*^unbound^_conform_	+14.75	2963
9	Δ*G*^unbound^_*ΘΦΨ*_	+6.80	—
	Total Δ*G*^0^_binding_	−26.32	5332

### Interaction of pep39 with the spike protein RBD

**Fig. 5:**
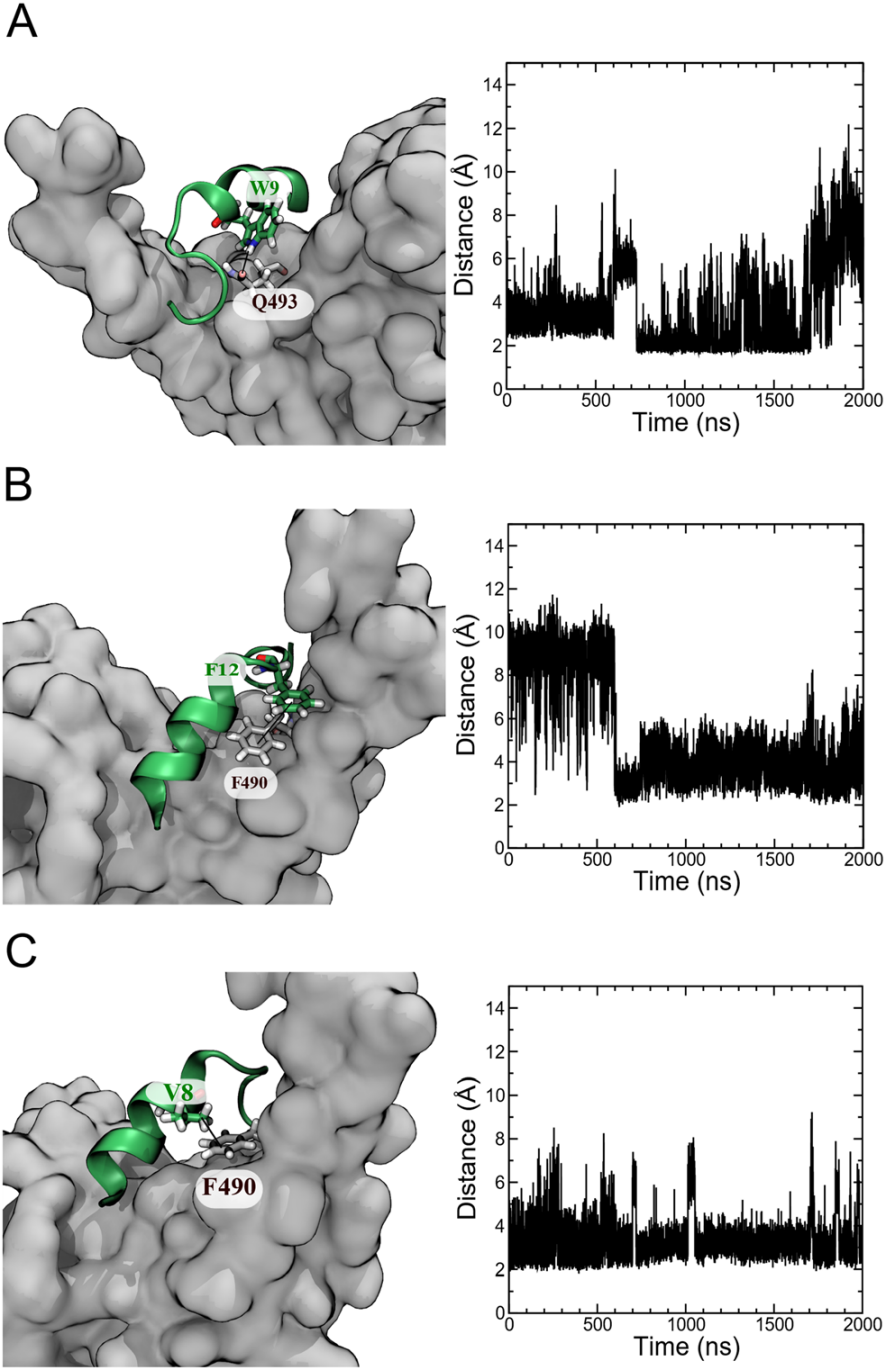
Atomic interactions between pep39 and the spike protein RBD. (A) H-bond involving the indole nitrogen of Trp9 and the sidechain amide oxygen of Gln493. (B) A π–π stacking interaction between Phe12 of pep39 and Phe490 of spike protein RBD. (C) A hydrophobic interaction between the aliphatic sidechain of Val8 and the aromatic ring of Phe490 of the spike protein RBD.

An analysis of unbiased MD simulation trajectories for pep39 bound to the spike protein RBD shows that pep39 occupies the region where the host protein ACE2 binds and makes contact with key residues of the binding site on the RBD like Lys417, Leu455, Phe490, Gln493, and Tyr505 on RBD.^31^ Fig. S4A† shows the distance of pep39 from the binding site (consisting of residues Trp353, Arg403, Lys417, Asn439, Val445, Leu455 Phe456, Gln493, Asn501, and Tyr505) is less than 3 Å for almost 1700 ns of the MD simulation. Some important interactions between pep39 and RBD are shown in the 3-dimensional view in Fig. S4B.† These include a hydrogen bond between the indole NH hydrogen of Trp9 and the sidechain amide oxygen of Gln493 of the spike protein RBD (Fig. 5A). A π–π interaction between the aromatic rings of Phe12 of pep39 and Phe490 of the spike protein (Fig. 5B) is also present for more than 500 ns out of 2000 ns of the simulation. The hydrophobic interaction between the aliphatic side chain of Val8 and aromatic ring of Phe490 of the spike protein was maintained for almost the entire simulation (Fig. 5C). Hence Phe490, one of the key residues in the binding site, was engaged with pep39 throughout the simulation. Overall, pep39 blocks the spike protein RBD surface where ACE2 protein binds.

### Experimental confirmation of the binding activity of pep39

#### Micro-cantilever-based method

**Fig. 6:**
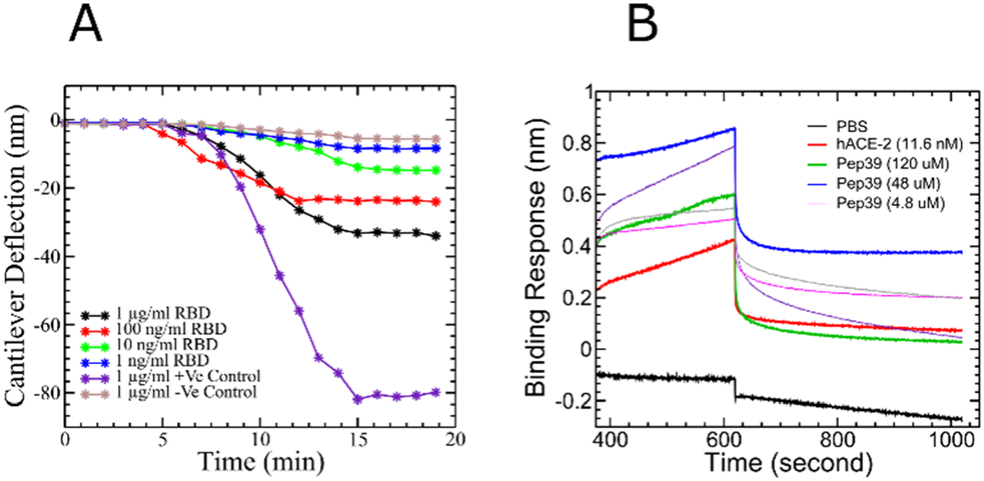
Binding kinetics study of pep39 and the spike protein RBD. (A) The deflection curve for the peptide pep39 immobilized on the cantilever in response to different concentrations of SARS-CoV-2 spike protein. Anti-S1 antibody served as a positive control and H1N1 protein, as a negative control. (B) The association and dissociation between immobilized spike protein RBD and different concentrations of pep39. Recombinant human ACE2 protein used as a positive control and PBS buffer as a reference blank.

On the basis of the deflection of the micro-cantilever, we were able to confirm the binding of pep39 on the surface of the spike protein RBD. The method involves the real-time monitoring of microcantilever bending (deflection) resulting from surface stress induced by a specific protein–protein interaction on the cantilever surface.^32,33^ The maximum deflection (signal) achieved was 33.27 nm for a 1 μg ml^−1^ target concentration, whereas the minimum signal of 8.46 nm was observed for the lowest concentration of the target analyte (1 ng ml^−1^). For the positive control (anti-spike protein antibody), a target concentration of 1 μg ml^−1^ was used, and we observed a maximum deflection of 81.88 nm. For the negative control, as expected, we measured the lowest deflection, 4.71 nm, which is not significant and can be considered as noise (Fig. 6A).

#### Bio-layer interferometry

BLI analyses the interference pattern of white light reflected from a biosensor tip with conjugated with protein molecules and an internal reference surface.^34,35^ The binding kinetics between the protein and peptide molecules are measured in real-time by detecting the shift in the interference pattern of the white light caused by binding and unbinding events at the surface of the protein immobilized biosensor tip. Human recombinant ACE2 was used as a positive control to validate the biosensor tip. In agreement with the previous reports, our BLI analysis has demonstrated a dissociation constant of 9 nM for binding between the spike protein RBD and ACE2.^36,37^ Concentrations of 120, 48, 24, 4.8, and 0.48 μM for pep39 were used to study binding activity with biotinylated recombinant SARS-CoV-2 spike protein RBD immobilized on high precision streptavidin (SAX) biosensor tips (Fig. 6B). The results were globally fit to determine the dissociation constant. The binding assay demonstrated that pep39 binds to spike protein RBD with *K*_D_ value of 570 ± 50 nM using BLItz Pro version1.1 software.

### Binding of pep39 to variants of the spike protein

**Fig. 7:**
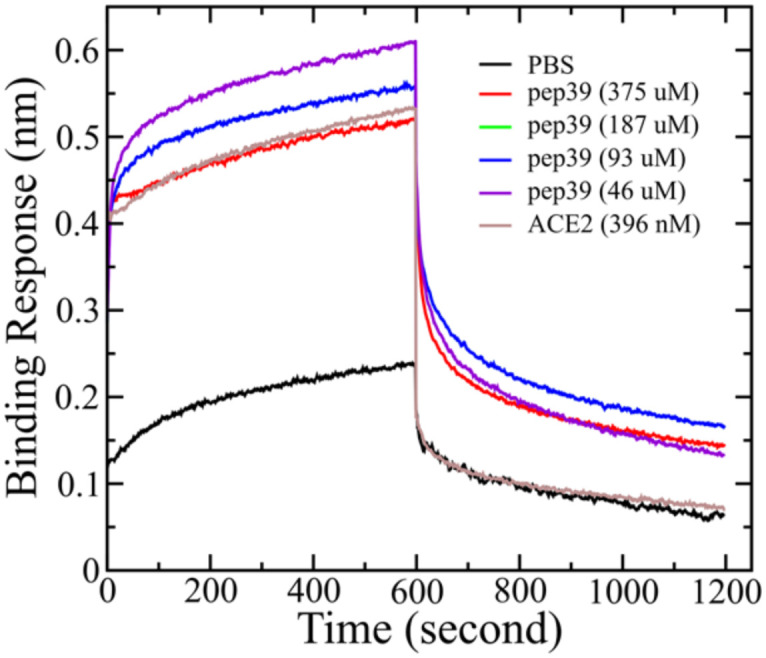
Binding kinetics study of pep39 and the delta variant spike protein. The association and dissociation between immobilized delta variant spike protein and various concentrations of pep39. Recombinant human ACE-2 protein was used as a positive control, and PBS buffer was the reference blank.

Like other RNA viruses, new variants of SARS-CoV-2 are emerging due to mutations. Variants with the mutations in the spike protein are major health concerns and alarming because they are more transmissible and capable of evading the immune response.^38^ Some variants like B.1.1.7 (alpha), B.1.351 (beta), P.1 (gamma), and B.1.617.2 (delta), which were first found in the United Kingdom, South Africa, Brazil, and India, respectively, were designated as variants of concern (VOC) by WHO.^39–41^ To evaluate the effect of pep39 on the VOCs, we performed almost 2 μs long MD simulations and MMGBSA calculation. Pep39 stays bound with all VOCs for the entire length of the simulation with significant binding affinity (Table 2). Among all VOCs, the delta variant caused a major wave of COVID-19 pandemic.^42–44^ Rui Wang *et al.* demonstrated that the delta variant would be a vaccine breakthrough variant due to its ability to disrupt the antibody-RBD complexes using a computational study.^45^ We performed BLI analysis to confirm the binding activity of pep39 with spike protein delta variant. Because the delta variant contains mutations in the N-terminal domain (NTD) and the receptor-binding domain (RBD), we immobilized the complete spike protein delta variant B.1.617.2 which has both subunits.^46^ The BLI assay reported that the ACE2 protein binds to spike protein delta variant B.1.617.2 with a *K*_D_ of 120 pM. As expected, the dissociation constant for the interaction of ACE2 and delta variant is more favorable for binding than the wild-type spike protein. Our results with the positive control agreed well with previous studies.^47^ To evaluate binding activity of pep39 with the spike protein delta variant, we used a broad range of pep39 concentrations (375, 187, 93, 46, 25, 5 and 500 μM), and the data were globally fit to determine the dissociation constant. The binding assay demonstrated that pep39 binds to spike protein delta variant with a *K*_D_ of 4.1 ± 1.4 μM using BLItz Pro version 1.1 (Fig. 7).

**Table 2:** MD simulation of pep39 with spike protein RBD variants and resultant MMGBSA binding free energy

Sl. no	Spike protein RBD variants	MD simulation run time (ns)	Estimate of binding energy by MMGBSA (kcal mol^−1^)
1	Alpha	1866	−41.8 ± 0.08
2	Beta	1845	−37.2 ± 0.09
3	Gamma	1759	−37.7 ± 0.09
4	Delta	2000	−41.3 ± 0.13
5	Omicron	2000	−30.2 ± 0.17

In previous studies, computational models of some anti-microbial peptides, cell-penetrating peptides conjugated with FDA-approved drugs, *de novo* design of peptides, and lipopeptides were described to prevent the entry of SARS-CoV-2, but experimental validation remains to be reported.^48–53^ Morgan *et al.* have reported binding activity of stapled peptides based on the ACE2 α1 helix, but these designed peptides did not prevent virus internalization.^22^ Curreli *et al.* also used a similar strategy to design double stapled peptides; their peptides showed activity in the range of IC50 1.9–4.1 μM, but effects on the spike protein variant have not been reported yet.^54^ A biochemically modified ACE2-targeting peptide derived from the spike protein showed significant inhibitory activity against ACE2 and spike protein association.^55^ Protein decoys, synthetic antibodies, and nanobodies have been explored to target the epitopes on the spike protein and achieved desirable binding affinity and neutralization effects.^56,57^ Recent report claims picomolar binding affinity of a synthetic antibody with the spike protein and its variants. Prophylactic and therapeutic effects were reported in the laboratory animals.^47^

These reports suggest that in order to inhibit the interaction between the spike protein and ACE2, a macromolecule is required as a drug. However, the production cost is the major limitation for antibodies and other macromolecular therapeutics and affordable options need to be explored. Our designed peptide exhibited a weaker binding affinity (500 nM for the original ancestral spike protein RBD and 4.1 μM for the delta variant) compared with some reported macromolecules, but still, this affinity is considerable. We performed biolayer interferometry experiments to evaluate the binding affinity of our designed peptide, pep39, towards the omicron (B.1.1.529) variant. Unfortunately, the results obtained with the omicron variant were not as promising as those observed with the original spike protein and the delta variant. Therefore, we made the decision to exclude the analysis of the recently identified sub-lineages of Omicron variants in the current study.^58,59^

### Effect of pep39 on the replication of the alpha variant of SARS-CoV-2 virus

**Fig. 8:**
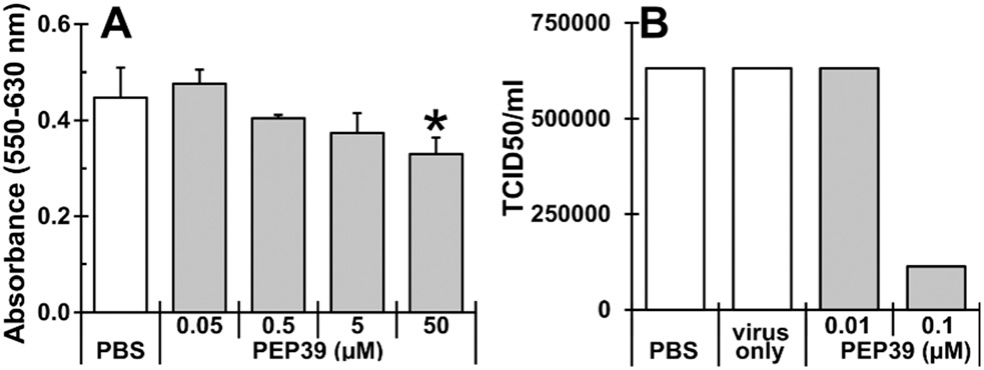
Evaluation of pep39 on its cytotoxicity and inhibitory effect on the replication of SARS-CoV-2 virus. (A) Cytotoxicity of pep39 on Vero E6 cells were evaluated using MTT assay (* indicates *P* < 0.05 compared with PBS). (B) Inhibitory effect of pep39 on SARS-CoV-2 viral replication in Vero E6 cells were evaluated by TCID50 assay.

To evaluate effect of newly designed peptide (pep39) on the replication of alpha variant of SARS-CoV-2 virus, cytotoxicity of pep39 was examined using Vero E6 cells, which express the ACE-2 protein and are commonly used for the *in vitro* screening of SARS-CoV-2 virus inhibitors.^60^ As shown in Fig. 8A, pep39 dose dependently inhibited cell growth at concentrations higher than 0.5 μM of the peptide and a significant inhibition was observed at 50 μM or above. As shown in Fig. 8B, viral replication was significantly attenuated at 0.1 μM pep39 (82.3% decrease as compared to PBS control). These results may suggest that designed peptide pep39 effectively inhibit binding between the viral S protein and ACE-2 on Vero E6 cells, thereby SARS-CoV-2 viral replication was significantly inhibited. However, the stability and membrane permeability of pep39 were not assessed in this study.

## Conclusions

Due to rapid mutation of SARS-CoV-2 and limitations of vaccination for immunocompetent patients, there is an urgent need for anti-SARS-CoV-2 therapeutics. The availability of an affordable drug is required to treat patients that belong to the lower income categories. Here we computationally designed stapled peptides to bind the SARS-CoV-2 spike protein receptor binding domain. The best performing peptide, pep39, was chosen for experimental validation. The physical binding experiments confirm that pep39 binds to the original spike protein and its delta variant with considerable binding affinity, while cell culture experiments suggest that it can inhibit replication of SARS-CoV-2. This peptide (pep39), or derivatives, could be considered as a potential candidate for COVID-19 therapeutics.

## Materials and methods

### Peptide modeling

We followed a peptide design protocol similar to the one we used in designing at cyclic peptide that binds CTLA4.^61^ We analyzed the X-ray structure of the complex between the receptor-binding domain of the spike protein and the ACE2 protein (PDB ID: 6LZG). To make peptide template, a fragment containing residues 26 to 42 were extracted from the ACE2 protein using VMD version 1.9.4 (Fig. 1B).^62^

### Flexible docking of template peptide with spike protein RBD and sequence optimization

Flexible peptide docking was performed using the FlexPepDock module of the Rosetta molecular modeling suite.^63^ Low energy conformations of the template peptide on the surface of spike protein RBD were generated. Based on the energy score and root mean square deviation (RMSD) from the initial conformation around 3–5 peptide poses were selected out of 500 for sidechain and sequence optimization. The optimization algorithm iterates between a conformational optimization phase, where it attempts to find the lowest energy conformation of the sidechains, and the design phase, where the algorithm applies the substitution of user-defined residues that attempt to lower the energy of optimized conformation. The conformational optimization phase includes rotational and translational movement of the entire peptide while keeping the carbon backbone fixed.^64–66^

### Bio-molecular system preparation

All the receptor protein models were built from the X-ray crystal structure of the spike protein RBD and human ACE2 complex (PDB ID: 6LZG). All structures were parameterized using the CHARMM36m force field^67^ and the input generator module of the CHARMM-GUI web-server.^68^ Glycosylation was performed, and disulfide bonds between residues 379–432, 488–480, 391–525, and 336–361 were added. Models of spike protein RBD variants alpha, beta, gamma, and delta were generated by making the mutations N501Y (alpha); K417N, E484K, N501Y (beta); K417T, E484K, N501Y (gamma) and E484Q, L452R (delta) respectively. The model of predicted omicron variant was obtained from a previous study.^69^ The peptide structures with the optimized sequence were added to the appropriate binding site on spike protein RBD using ZDOCK, a protein-peptide docking algorithm.^70^ Each protein–peptide complex was solvated using ≈9000 molecules of water (TIP3P water model), and 150 mM of sodium chloride ions were added, with additional ions to neutralize the system.^71^ The overall volume of the system was (80 Å),^3^ and the number of atoms was ≈50 000.

### Molecular dynamics simulation and MMGBSA calculation to estimate binding free energy

All molecular dynamics simulations were performed in the NPT ensemble using the program NAMD version 2.13,^72^ where a Langevin thermostat was applied to maintain a temperature of 310 K and the Langevin piston barostat algorithm was used to maintain a pressure of 1 standard atmosphere.^73,74^ Interatomic forces were defined by CHARMM36m force field. The Lennard-Jones interaction between pair of atoms calculated using a smooth 10–12 Å cut off distance and electrostatic interactions were implemented using the particle mesh Ewald (PME) with 1.2 Å grid spacing.^75,76^ Energy minimization for each system was performed for 1 ns and followed by a production simulation for 2 μs without applying any restraints on the atoms. The NAMD Colvars module was applied to terminate the MD simulation if the conformation of the peptide changed more than cut off value (RMSD >15 Å) from the initial bound pose.^77^ At every 200 ps, the configuration of the biomolecular system was collected for further analysis.

The binding free energy for each frame of the MD simulation for each system was estimated using the molecular mechanics generalized Born surface area (MMGBSA) method as described in the eqn (1).^78,79^ The implementation of this method involves an implicit solvent with a dielectric constant of 78.5 and a surface tension of 0.00542 kcal (mol^−1^ Å^−2^) to estimate the solvation free energy of the extracted protein, peptide, and protein–peptide complex for each frame of the MD trajectories.

1 Δ*G*^GBSA^_binding_ = Δ*G*^GBSA^_protein : peptide_ − Δ*G*^GBSA^_protein_ − Δ*G*^GBSA^_peptide_

### Stapling of better performing peptides to maintain the favourable binding conformation

Peptides were shortlisted based on the MMGBSA score. The most favorable configuration of the peptides was extracted. To maintain this most favorable conformation, side chains that did not make contact with the binding interface of ACE2 were selected for the stapling process (from residue *i* to residue *i* + 4). Selected sidechains of aliphatic amino acids were linked using a propene staple. Side chains of charged residues (such as Asp and Lys) were connected using extra peptide bond. CHARMM-format patches for applying these staples are included in the official CHARMM distribution.^80^ MD simulations and MMGBSA calculations were performed for each system containing a stapled peptide and the spike protein RBD.

### Absolute binding free energy calculation by geometric route

The configuration corresponding to the lowest MMGBSA energy was extracted and used as an input for the binding free energy estimator (BFEE) plugin of VMD version 1.9.4.^81^ BFEE subdivides the binding free energy calculation into different subprocesses (Table 1). The key idea is that calculating the free energy for unbinding the peptide is much more efficient if artificial restraints are applied to the conformation and orientation of the peptide relative to the receptor.^82^ In fact, calculation is not feasible without these restraints. However, such restraints bias the result, so their effect must be calculated and removed to obtain the unbiased binding free energy. First, the free energy cost of releasing these restraints from the bound peptide is determined by calculating a potential of mean force along each restrained coordinate using the extended adaptive biasing force (eABF) method as implemented in the Colvars module.^83,84^ This comprises subprocesses 1–6 in Table 3. Next the free energy of unbinding the re-strained peptide is calculated (subprocess 7). The free energy cost of applying the restraints to the unbound peptide in solution are then calculated (subprocesses 8 and 9). The cost of applying the orientational restraints to the bound peptide can be computed analytically owing to the isotropy of the unbound peptide in solution. The dissociation constant is calculated using eqn (2) (see Fu *et al.* for more details):

2 *K*_d_ = exp{−*β*(Δ*G*_conform_ + Δ*G*_*Θ*_ + Δ*G*_*Φ*_ + Δ*G*_*Ψ*_ + Δ*G*_*θ*_ + Δ*G*_*ϕ*_ − *k*_B_*T* ln(S*I*C^0^) + Δ*G*^unbound^_conform_ + Δ*G*^unbound^_*θϕΨ*_)}

where 
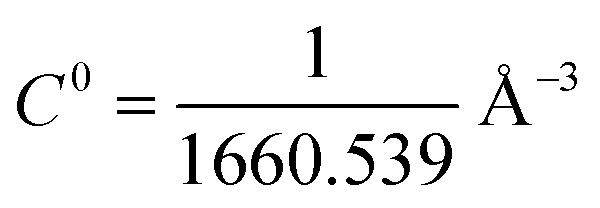
 is the standard 1 mol L^−1^ concentration, 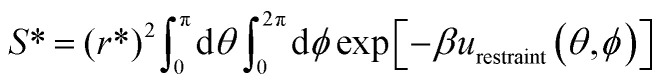 and I* = ∫d*r* exp{−β[*w*(*r*) − *w*(r*)]}, and *w*(*r*) is the radial potential of mean force for extracting the restrained peptide from the protein calculated in subprocess 7.

**Table 3:** Subprocesses for rigorous free energy calculation

Stage	System	Free-energy term	Description
1	Protein–ligand	Δ*G*_conform_	Release RMSD restraint on bound peptide
2	Protein–ligand	Δ*G*_*Θ*_	Release Euler angle *Θ* restraint on bound peptide
3	Protein–ligand	Δ*G*_*Φ*_	Release Euler angle *Φ* restraint on bound peptide
4	Protein–ligand	Δ*G*_*Ψ*_	Release Euler angle *Ψ* restraint on bound peptide
5	Protein–ligand	Δ*G*_*θ*_	Release polar *θ* restraint on protein–peptide vector
6	Protein–ligand	Δ*G*_*ϕ*_	Release polar *φ* restraint on protein–peptide vector
7	Protein–ligand	−*k*_B_*T* ln(S*I*C^0^)	Bind peptide with above restraints to protein
8	Ligand only	Δ*G*^unbound^_conform_	Apply conformational restraint to unbound peptide
9	Ligand only	Δ*G*^unbound^_*θϕΨ*_	Apply orientational restraints to unbound peptide (computed analytically)

### Binding confirmation of the designed stapled peptide pep39 by the micro-cantilever method

We purchased silicon cantilevers from Nanoworld Inc., and the SARS-CoV-2 spike protein RBD, the antibody for the spike protein RBD and influenza H1N1 hemagglutinin protein were procured from Sino Biological Inc; and 1-ethyl-3-(3-dimethyl aminopropyl) carbodiimide and sulfo-NHS from ThermoFisher Scientific. The designed stapled peptide pep39 was commercially synthesized from LifeTein LLC (An HPLC chromatogram and MS spectrum graph, illustrating the purity and molecular weight of the peptide, is presented in Fig. S5†). The cantilever tips were plasma cleaned before immobilization. The microcantilevers were covalently immobilized by EDC-NHS chemistry using 100 μM of the designed staple peptide (referred to as pep39) prepared in PBS and 0.05% BSA (pH = 7.4) solution.^85^ The immobilized micro-cantilevers were brought into a microfluidic chamber containing the spike protein RBD. Antibodies to the spike RBD protein and the influenza H1N1 hemagglutinin protein (1 μg ml^−1^ each) were used as positive and negative controls, respectively. All experiments were conducted on a Bruker Bioscope Resolve liquid imaging system at a constant temperature, and cantilever deflection was measured using an in-built optical detector.

### Binding assay of pep39 by the bio-layer interferometry

The biotinylated recombinant SARS-CoV-2 spike protein RBD with His-tag, recombinant SARS-CoV-2 spike B.1.617.2 with His-tag and recombinant human ACE-2 protein were purchased from R&D Systems, Inc. High precision streptavidin (SAX) and anti-penta-His (high precision streptavidin (SAX)) biosensors obtained from the Sartorius Corporation. A solution of the spike protein at a 1 μg ml^−1^ concentration was loaded onto the corresponding hydrated biosensors. Each labeled biosensor was placed in different molar concentrations (120, 48, 24, 4.8, and 0.48 μM) of pep39, and association was measured for 120 seconds and followed by dissociation with PBS for 120 seconds. PBS buffer alone and 44 μg ml^−1^ human ACE-2 protein were used as a reference and a positive control, respectively. Experimental association and dissociation constants from all experiments were globally fitted using a 1 : 1 binding model to measure the dissociation constant *K*_d_ using the built-in software BLItzPro version 1.1. All binding assays were performed on the FortéBio BLItz instrument.

### Evaluation of the effect of pep39 against viral replication in the cell

Inhibition of viral replication was evaluated by incubating micromolar concentrations of peptide with a constant concentration of SARS-CoV-2 virus. Controls included peptide without virus as well as PBS, evaporated PBS and culture media with or without virus. The SARS-CoV-2 alpha variant of concern (VOC) SARS-CoV-2/human/USA/CA_CDC_5574/2020 lineage B.1.1.7 strain (BEI item #: NR-54011) was acquired from BEI Resources (Manassas, VA, USA), and a passage 1 virus stock was used for these studies. The stock virus was sequenced on the Illumina MiSeq and was found to be in consensus with the original BEI strain [GISAID accession number: EPI_ISL_751801 (CA_CDC_5574/2020)]. Peptide, PBS, evaporated PBS, or culture medium was mixed with virus or culture media. Virus was tested with peptide at 400 tissue culture infectious dose 50 (TCID50) or approximately at a multiplicity of infection (MOI) of 0.01. After 15 minutes incubation at room temperature, 100 μl of each sample mixture was added per well of a 96-well plate of Vero E6 cells (ATCC). Due to the cytotoxicity of the pep39 in Vero E6 cell culture higher than 0.5 μM, TCID50 assay was carried out only low concentrations of the peptide (0.01–0.1 μM). Quadruplicate determinations were performed. After 48 h, cell culture supernatants were collected for endpoint virus titration (TCID50) based on cytopathic effect (CPE) by performing serial 10-fold dilutions on 96-well plates of Vero E6 cells.

### Effect of pep39 on the growth of Vero E6 cells

Vero E6 cells were seeded into 96-well plate. After 24 h, the cells were treated with 0.05–50 μM pep39. At 48 h after treatment, the cell viability was evaluated using 3-(4,5-dimethylthiazol-2-yl)-2,5-diphenyltetrazolium bromide (MTT) assay as previously described.^86^

### Statistical analysis

All values are expressed as the mean ± standard deviation of mean. For all *in vitro* experiments, statistical significance was assessed by unpaired *t*-test or ANOVA followed by Tukey's test. All experiments were conducted with multiple sample determinations with several samples. Statistical significance was set at *P* < 0.05.

## Data availability

The dataset provided for the manuscript entitled “*De novo* design of a stapled peptide targeting SARS-CoV-2 spike protein receptor-binding domain” are available on (https://doi.org/10.5281/zenodo.7844171). The dataset encompasses all the required files to execute, and analysed simulations of a designed stapled peptide (Pep 39) attached to spike protein receptor binding domain in the most stable binding configuration, illustrated in Fig. 3 of the paper. The dataset is composed of molecular model structure files in NAMD psf format, force field parameter files in CHARMM format, initial atomic coordinates in PDB format, NAMD configuration files, NAMD output files (which consist of restart files in binary NAMD format), and trajectories in dcd format (down sampled to 10 ns per frame). To manage the analysis, there are shell scripts (that work with Bash) that invoke VMD Tcl scripts. These scripts and their output are also contained in the dataset.

## Conflicts of interest

The J. A. R. laboratory received support from Tonix Pharmaceuticals, Xing Technologies, and Zoetis, outside of the reported work. J. A. R. is inventor on patents and patent applications on the use of antivirals and vaccines for the treatment and prevention of virus infections, owned by Kansas State University, KS.

## Supplementary Material

MD-014-D3MD00222E-s001
