# Supplementary material for: De novo design of a stapled peptide targeting SARS-CoV-2 spike protein receptor-binding domain
Source: RSC Med Chem. 2023 Jul 31;14(9):1722–33. doi: 10.1039/d3md00222e (PMC10507807; doi:10.1039/d3md00222e)
Supplement: MD-014-D3MD00222E-s001 [file MD-014-D3MD00222E-s001.pdf]

Electronic Supplementary Material (ESI) for RSC Medicinal Chemistry.
This journal is © The Royal Society of Chemistry 2023

Supplementary material for

De novo design of stapled peptide targeting SARS-Cov-2 spike protein receptor-binding domain

Ravindra Thakkar[1*], Dilip K Agarwal[2], Chathuranga B Ranaweera[3], Susumu Ishiguro[1], Martin Conda-Sheridan[4], Natasha N. Gaudreault[5], Juergen A. Richt[5], Masaaki Tamura[1] and Jeff Comer[1].

[1], Department of Anatomy & Physiology, Kansas State University College of Veterinary Medicine, Man-hattan, Kansas, United States

[2], Department of Material Science and Engineering and NUANCE Center, Northwestern University, Evanston, Illinois, United States

[3], Department of Medical Laboratory Sciences, General Sir John Kotelawala Defense University, Colombo, Sri Lanka

[4], Department of Pharmaceutical Sciences, College of Pharmacy, Universit

y of Nebraska Medical Center, Omaha, Nebraska, United States

[5], Department of Diagnostic Medicine & Pathobiology, Kansas State University College of Veterinary Medicine, Manhattan, KS.

Corresponding Author: Ravindra Thakkar
Department of Anatomy & Physiology, College of Veterinary Medicine,
Kansas State University, Manhattan, Kansas, United States
Phone: (785) 210-6108
E-mail: ravithakkar@vet.k-state.edu

Table–S1: Optimized peptide sequences with Rosetta design score, length of a simulation in which the peptide stayed bound and estimated binding free energy by MM-GBSA method.

ID	Sequence	Design score	Time Bound (ns)	Rough Estimate of Binding Energy by MMGBSA (kcal/mol)
1	NSLKELTEKEATDKYYK	-232.95	66.6	+10.845 ± 0.586
2	SIEELIKKNKKTIEKYK	-99.762	321	-17.274 ± 0.198
3	EDLKKWAKQLEELKYK	-237.75	198	-24.442 ± 0.369
4	YESLLRFLTKLKDDKYK	-240.42	67.4	-12.467 ± 0.396

5	ATEEETKKAKAEDKKYK	-239.59	193.4	-14.902 ± 0.334
6	EEYEKMLESLKELKHYK	-105.68	183.4	-29.737 ± 0.351
7	YTFKEKTDHEATDKYYK	-230.47	13.6	-03.171 ± 0.669
8	YTFEVKEKHKRDIEKYK	-91.879	164.6	-12.253 ± 0.322
9	YTFLKKWAHQAEELKYK	-229.85	231.2	-36.851 ± 0.518
10	YTFLEKFLHKLKDKKYK	-236.83	76.2	-12.328 ± 0.405
11	YTFEAKVKHEQEDKKYK	-235.7	12.8	-05.877 ± 0.730
12	YTFEAKKKHEEELRHYK	-92.738	1032.8	-28.023 ± 0.178
13	SEDLAKWAETLERLKYK	-230.58	131.6	-21.556 ± 0.449
14	NVEKRTKEAKAEDSHYK	-234.14	1572.2	-30.562 ± 0.164
15	SIEELIKRLKKTEEKYK	-220.31	1148.6	-24.423 ± 0.131
16	YDDLEKWKKQLEELKYK	-229.13	31.2	-10.648 ± 0.376
17	EEEEKTKKILTELAKYK	-218.78	265.4	-24.564 ± 0.311
18	STEDEEKKAKAEDSLYK	-238.36	165.8	-12.937 ± 0.340
19	STDQEYKKAVEHDRKYK	-233.12	13.6	-07.521 ± 0.551
20	PDDLKIWAQILLEAFYK	-232.02	2000	-34.147 ± 0.097
21	SIEDKIKKAKAVVAKYK	-229.62	245.2	-18.212 ± 0.289
22	QTQDEWKKAVEEEKKYK	-234.59	155.8	-29.358 ± 0.118
23	STDDEKKKALAEDQQYK	-236.3	1039.4	-17.186 ± 0.152
24	SELEKALKALSSLSTYK	-230.04	351.8	-22.292 ± 0.182
25	EEEEKKKTAKKELDTYK	-226.02	274.6	-25.472 ± 0.336
26	YEELKKWAKTLEEAKYK	-228.17	239.4	-16.526 ± 0.170
27	SLEDLIEEAKRKIEKYK	-219.24	371.2	-16.070 ± 0.169
28	TFLEEWLKQKEELKLK	-172.52	2000	-28.882 ± 0.092
29	TFLELWKRQAKDLRYK	-181.95	435.4	-21.133 ± 0.240
30	TFLEKWLKQADDLKNK	-175.92	111	-22.651 ± 0.477
31	DELEITADKLRQAMEK	-233.77	365	-14.595 ± 0.178
32	DELEVLAEQLREALAK	-230.03	83.8	-15.091 ± 0.310
33	DPWEIWAKLLKESRDK	-237.51	1421	-29.714 ± 0.085
34	GTEWLYKVWKAFVENMS	NA	1350.8	-29.688 ± 0.122
35	STEWLKKVHWKFHAKFS	NA	29.6	-03.422 ± 0.468
36	GTEEIYKRWATVMKKKK	NA	880.2	-11.344 ± 0.098

37	GTEWLKKVWEAFVKNMS	NA	189.4	-28.698 ± 0.335
38	GTEEIYLWMVTVHKQKK	NA	151	-17.313 ± 0.471
39	STEYLKRIFWMFHEYES	NA	342	-29.252 ± 0.217
40	NVEKRTKEAKAEDSHYK	NA	1253.8	-45.021 ± 0.153
41	QTQDEWKKAVEEEKKYK	NA	1014.12	-23.381 ± 0.116

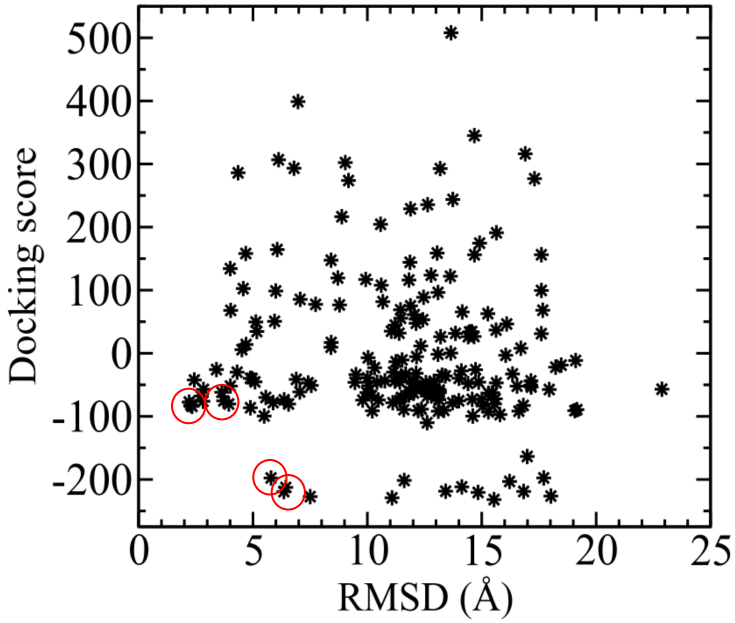

Figure–S1 Docking score versus RMSD plot for docked poses of the template peptide on the spike protein RBD surface.

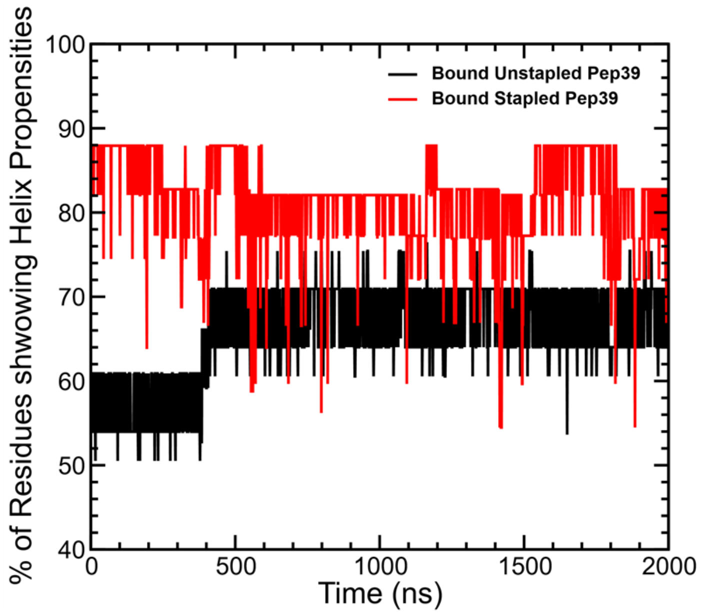

Figure–S2 The percentage of residues showing α Helix propensities for stapled and unstapled peptide in the bound state.

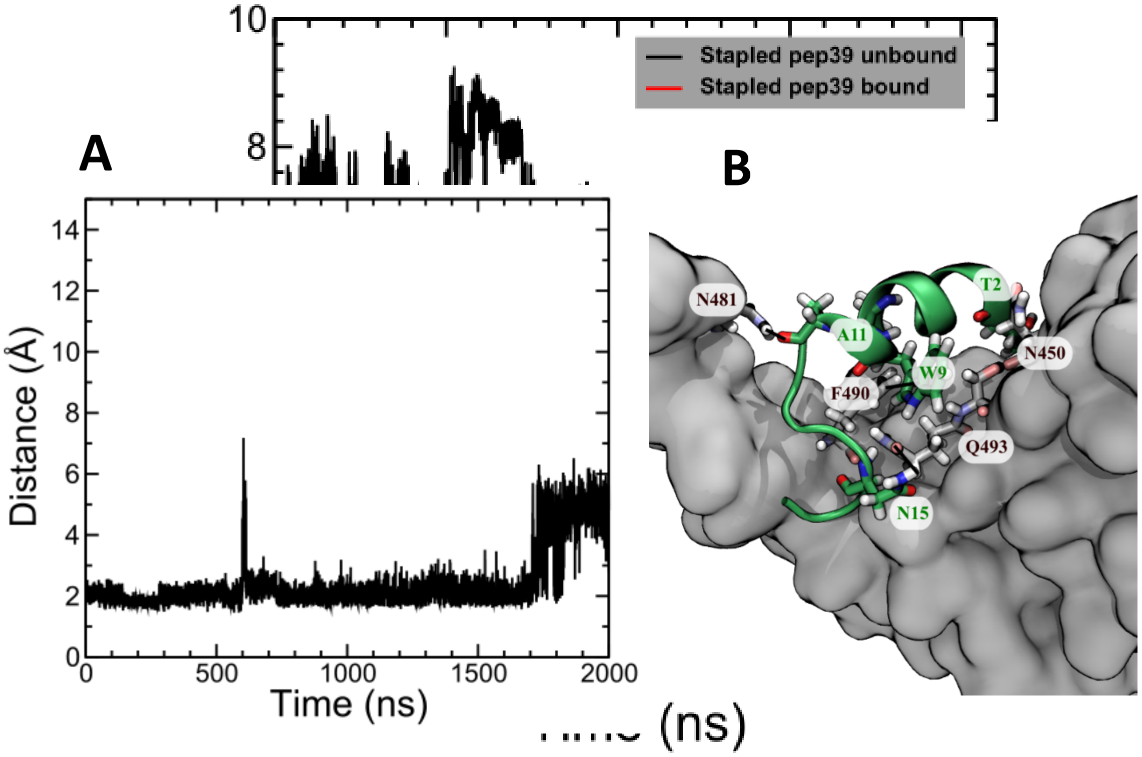

Figure–S4 (A) Distance of the pep39 from the ACE2 binding site on the spike protein RBD. **(B)** 3D view of the interaction between designed peptide and spike protein RBD.

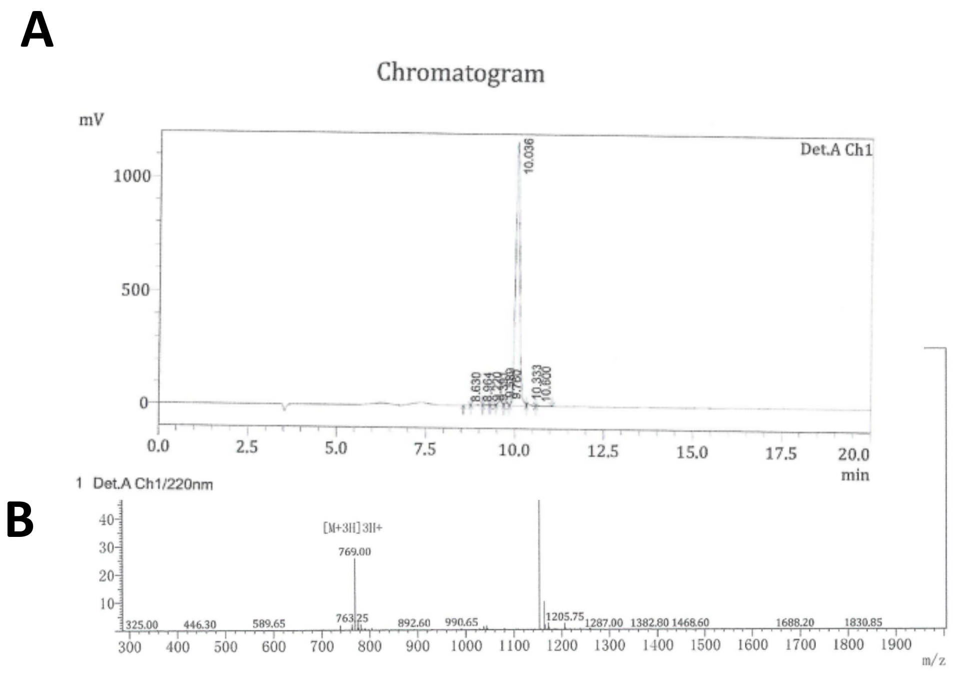

Figure–S5 (A) HPLC chromatogram and **(B)** MS spectrum graph of the stapled peptide [illegible]

Table–S2: Optimized peptide sequences with time remaining bound during the MD simulation for stapled and unstapled peptides and the estimated binding free energy by MM-GBSA method

Sl. No	ID	Time Bound (ns)		Rough Estimate of Binding Energy by MMGBSA (kcal/mol)		Absolute Binding Free Energy (Stapled) (kcal/mol)
		Stapled	Unstapled	Stapled	Unstapled	
1	Ngu-123	2000	189.4	-24.958 ± 0.063	-24.958 ± 0.063	NA
2	Ngu-335	1547	342	-28.557 ± 0.106	-29.252 ± 0.217	NA
3	Ngu-129	2000	2000.0	-29.000 ± 0.072	-34.147 ± 0.097	NA
4	Ngu-129a	371		-18.303 ± 0.144		NA
5	Ngu-29	2000	1253.8	-33.970 ± 0.119	-45.021 ± 0.153	-05.02 ± 15.37
6	Ngu-29a	162		-14.611 ± 0.901		NA
7	Ngu-29b	2000		-44.746 ± 0.122		-13.31 ± 01.63
8	Ngu29c	77.6		-12.620 ± 0.455		NA
9	Ta-93	1398.8	198.0	-33.945 ± 0.151	-36.851 ± 0.518	-17.41 ± 03.10
10	Ta-200	18.2	1421.0	-13.137 ± 0.491	-29.714 ± 0.085	NA
11	Ta-196	415.4	1032.80	-21.832 ± 0.213	-28.023 ± 0.178	NA
12	Ta-26	189.8	2000	-29.407 ± 0.259	-28.882 ± 0.092	NA
13	pep-39	2028.8	1350.8	-33.936 ± 0.071	-29.688 ± 0.122	-26.32 ± 08.64
14	pep-39a	2000		-28.884 ± 0.055		NA
15	pep-39us	1222		-27.037 ± 0.076		NA
